# Disruption of the Colonization Resistance Syndrome in Humans in Altered Habitats and Its Prevention

**Published:** 2014

**Authors:** V. K. Ilyin, N. V. Kiryukhina

**Affiliations:** Institute of Biomedical Problems, Russian Academy of Sciences, Khoroshevskoe shosse, 76A, Moscow, Russia, 123007

**Keywords:** autostrains, autoprobiotics, dysbacteriosis, altered habitat, disruption of colonization resistance

## Abstract

Exposure of human subjects to environments with modified parameters is associated with reduced colonization resistance of the intestine and epithelial tissue, which leads to dysbiotic changes. Probiotics – preparations based on protective microflora – are used to correct dysbacteriosis of different etiologies and localizations. However, the effectiveness of probiotics largely depends on the adhesive ability of a probiotic strain and lack of competitive relations with the indigenous microflora, which can be achieved by individual selection of a preparation. We propose to use autochtonous microflora as a probiotic drug to optimize the prevention and treatment results. A personalized approach to probiotic selection will improve therapy efficiency and reduce the risk of adverse effects in each individual patient.

## INTRODUCTION

Human exploration of space, oceans, and the Earth’s core leads to the creation of bio-isolated artificial human ecosystems with modified habitat parameters [1]. It substantially alters the phylogenetically established relationships between commensals of the human–microflora ecosystem. In humans, this manifests itself as the disruption of the colonization resistance syndrome**.** It is, therefore, evident that in order to develop a strategy of environmental approaches to disease prevention in humans in extreme habitats we need to study the state of natural colonization barriers against infectious agents in the human body.

Van der Waaij [2] defined “colonization resistance” as “resistance which a potentially pathogenic microorganism encounters when it tries to colonize a ‘landing site’ on the mucosa in one of the three tracts that have an open communication with the outside world: respiratory, urinary and digestive.” van der Waaij identifies two main barriers responsible for resistance to infections in humans: the barrier formed by commensal microflora and the barrier provided by factors of cell-mediated and humoral immunity. V.M. Bondarenko [3] additionally mentions the epithelium of mucous membranes as a natural barrier, as its physiological state largely defines its permeability to causative agents. Studies by Noble [4] allow us to add skin tissue to the list of colonization barriers.

The first and main barrier against colonization, the barrier formed by microbial associations of commensals in a human body, deserves a more detailed analysis. The system of relationships both within such associations and between associations and the host is quite complex. When a person is infected, a causative agent enters the host organism as a population of genetically heterogeneous cells through food, water, particles of drop or dust aerosols, etc. The primary focus of infection is formed as a causative agent displaces normal host microflora and colonizes the new habitat. Adhesion, colonization, and subsequent propagation of the agent that synthesizes toxic compounds leads to pathomorphological changes in the host; in the case of opportunistic microorganisms, it is characterized by a lack of specificity and mosaic disorders in various organs and tissues. Impairment of the overall resistance of the organism and lower levels of protective microflora results in an increased population of opportunistic microorganisms, which can translocate to other biotopes [5]. Endogenous infections etiologically caused by autochtonous microflora develop along a similar pathway.

According to modern beliefs, the natural microflora of any biotope can be divided into permanent (resident) and accidental (transient) based on its origin. Resident microflora includes microorganisms specific to a certain biotope, whereas the transient one consists of exogenic microorganisms. The composition of the resident microflora of a biotope is relatively stable; however, the physiological importance of its microorganisms differs substantially. Therefore, resident microflora is further subdivided into an obligatory and a opportunistic one.

Obligatory microflora is the main component of any microbiocenosis; it prevents colonization of the biotope by random microorganisms and is involved in fermentation and immunity stimulation processes. Thus, obligatory microflora in the large intestine includes bifidobacteria, lactobacilli, typical collibacilli, peptostreptococci, eubacteria, and most bacteroid and enterococci species [6]. The natural microflora of the digestive tract has important physiological functions: it enables colonization resistance of the mucosa; stimulates the formation of the immune system in newborns; maintains the immune status in adults via muramyl peptides of bacterial cell walls and other adjuvant-active macromolecules; participates in metabolic processes (secretion of the enzymes involved in the protein, lipid, nucleic and bile acid metabolism); maintains the electrolyte balance and synthesis of vitamins B, K and D; regulates the gaseous environment in the intestine; is involved in the biochemical processes of digestion (fermentation of food substrates, regulation of the motor- evacuation function of the intestine); and it inactivates toxic exogenous and endogenous products by biotransformation and biodegradation.

A sufficient number of resident microorganisms attached to the intestinal walls prevent the propagation of pathogenic agents, their invasion of enterocytes and passage through the intestinal wall; this is achieved by the formation of an environment with pH values adverse to extraneous microflora in the biotope, as well as by the production of bacteriocins (antibiotic substances) and the deprivation of competing microorganisms of nutrients and adhesion sites.Beneficent metabolic activity also includes the production of vitamin K, biotin, niacin, pyridoxine and folic acid; the hydrolysis of bile salts and cholesterol; regulation of its levels; and hormone re-circulation. The lack of a favorable microflora in the intestinal microbiocenosis leads to a disruption in the recirculation of the estrogenes secreted in the gastrointestinal tract (GIT) with bile and the development of corresponding pathological disorders in the female reproductive system. Normally, functioning resident microflora controls the toxin production in the intestine, as well as prevents their over-expression and penetration into the bloodstream. Resident microflora has detoxifying and proteolytic properties, which allow it to metabolize endotoxins, allergens, and antigens in the intestine by proteolysis. It also involves absorption of partially digested proteins in the intestine, including those associated with the development of food intolerance and accompanying skin disorders. A disruption in the microbiocenosis allows these substances to enter the bloodstream.

The detoxifying and protective role of indigenous microflora in preventing the adverse effects of radiation, chemical impurities in food, carcinogenic factors, toxic exogenous substrates, unfamiliar and exotic food, and contaminated water is also noteworthy. This occurs by the stimulation of the immune response and improvement of non-specific immune resistance: potentiation of the production of interferons, interleukins and enhancing the phagocytic abilities of macrophages.

Let us describe the main groups of protective microflora used to produce popular modern probiotic preparations.

Bifidobacteria constitute the main group of intestinal bacteria; they amount to 25% of the entire intestinal microbial population in adults and 95% in newborns. Bifidobacteria produce acetic and lactic acids. Subsequent development of an acidic environment induces an antibacterial effect. Bifidobacteria are capable of releasing metabolic products that can directly inhibit the development of opportunistic Gram-positive and Gram-negative pathogens. Bifidobacteria convert potentially toxic ammonia (or amines) into NH_4_ ions that are unable to penetrate through mucosa and reach the bloodstream. Furthermore, these bacteria do not form aliphatic amines, hydrogen sulfides, or nitrites. Bifidobacteria produce vitamins (mostly group B ones), as well as digestive enzymes, such as casein phosphatase and lysozyme. Bifidobacteria restore the normal intestine microflora after antibiotic therapy [6].

Enterococci, previously classified as group D streptococci, are a large group of bacteria belonging to the genus *Enterococcus*, which includes the *E. faecalis*,* E. faecium, E. avium, E. casseliflavus*, *E. durans*,* E. gallinarum*,* E. raffinosus*, *E. irae*, *E. malodoratus* and *E. mundtii *species. *E. faecalis*, *E. faecium*, *E. gilvus* and *E. pallens *were identified in human clinical isolates. Enterococci are detected in newborns as early as in the first days of their lives; in breastfed babies under 1 year, their levels vary between 10^6^ and 10^7^ CFU/g. In formula-fed babies enterococci levels can reach 10^8^–10^9^ CFU/g. The enteroccoci levels in the intestine of a healthy human remain consistent and reach 10^7^–10^8^ CFU/g of feces. Enterococci are present in almost every intestine section. The main properties of enterococci include involvement in the synthesis of vitamins and metabolism of sugars (lactose); immunostimulation (maintenance of the level of broad-spectrum cytokines); high antagonistic activity against staphylococci, listeria and collibacilli via the production of bacteriocins; anti-inflammatory activity; and high resistance to environmental factors (temperature, pH). Normally, the levels of enterococci in the intestine do not exceed the overall level of collibacilli [6].

Lactobacilli also constitute a substantial part of protective microorganisms’ population in most human biotopes. Lactobacilli are Gram-positive rode-shaped bacteria, opportunistic anaerobes. Lactobacilli differ in their requirements for nutrients and growth factors. Lactobacilli exhibit proteolytic activity mediated by the activity of the proteases and peptidases they produce; their lipolytic properties allow them to digest milk fat and some triglycerides; they also synthesize DNAse and/or RN Ase and pseudocatalase through exonuclease activity; and they produce enzymes that ferment hexoses, disaccharides and polysaccharides. The antagonistic activity of Lactobacilli is mediated by their extensive ability to produce an acidic environment as well as by the production of antibiotic compounds (such as acidophilin – *Lactobacillus acidophilus*, lactolin –* L. plantarum*, brevin – *L. brevis*), hydrogen peroxide, and lysozyme [6].

The mechanisms of colonization resistance can be divided into a direct and indirect activity. The direct mechanisms include the production of inhibitors that interrupt the metabolism of pathogenic and opportunistic bacteria by bacterial strains, competitive relationships with pathogenic bacteria for nutritious substrates and adhesion sites, direct degradation of toxins, anti-endotoxic activity, and the prevention of microorganism translocation to other parts of the body. The indirect effects include stimulation of the immune system, stimulation of mononucleocytes, induction of interferon, inhibition of bile acid conjugation, etc. The observed variety of colonization resistance mechanisms naturally implies a large number of variants, particular combinations under certain circumstances, which define the state of colonization resistance that most likely depends on the microflora amount and quality and its habitat.

The aforementioned functions, beneficial to human health, are stable if both a quantitative and qualitative consistency of microflora is maintained. It is also natural that not all the beneficial functions of microflora are exhibited in all biotopes or are exhibited to an equal extent. They depend on the anatomical, physiological, and biochemical features of a biotope (i.e., gastrointestinal, urological tracts, skin or respiratory tract, etc).

## DISRUPTION OF COLONIZATION RESISTANCE UNDER THE INFLUENCE OF EXTREME FACTORS

Extreme conditions disrupt the barrier functions of the body associated with normal functioning of the skin, intestine, and mucous membranes, which come in direct contact with the environment [7]. Stress (psychoemotional or physical) has been shown to cause the activation of endogenous microflora in the intestine, penetration of bacteria into the bloodstream, and subsequent excretion through the urinary tract [8]. If the intensity of the factors affecting either directly or indirectly the fixation, survival, and functioning of normal microflora exceeds the capacity of the compensatory mechanisms of the host-microflora ecosystem, this will induce microenvironmental disruptions; their type, degree of manifestation, and duration will depend on impact dose and length. The ability to resist noci-influence depends on many factors, which characterize the state of the organism. Studies involving astronauts, divers, and athletes have revealed symptoms of secondary immunodeficiency and impairment of the regulatory mechanisms. The response of each individual depends on his genetic and immunological potential, as well as on the state of his microbiocenosis [9, 10].

Nowadays, the disruption of colonization resistance is considered to be a pathological condition that manifests in people holding “extreme jobs”: astronauts, scuba divers, submariners, compressed air workers, etc. Dysbacteriosis is one of the most important manifestations of the disruption of colonization resistance, which is characterized by a loss or reduction in the levels of some obligatory representatives of normal microflora, increased incidence of the concentration of representatives of opportunistic microflora, and possible appearance of bacterial strains atypical of a certain biotope.

The entire combination of factors affecting humans or animals during a space flight or prolonged exposure to hyperbaric conditions is quite unique; therefore, no adaptive measures against it have been developed through evolution [11]. In addition, the data suggest that such factors as nervous and functional stress, hypokinesia, extreme exercise load, prolonged stay in isolated conditions with modified parameters of the gaseous environment and microclimate also contribute to the development of dysbacteriosis [12]. The same study shows that nervous and functional stress results in a reduced number of bifidobacteria and lactobacilli, in some cases up to their complete eradication. Changes in aerobic microflora leading to increased concentration of some representatives also occur in the case of extreme exercise load. Pronounced changes in microflora also occur in people after they stay in a diving chamber with an altered gaseous environment and microclimate. These are common changes in the intestinal microflora resulting from exposure to extreme situations. The development of dysbacteriosis is triggered by a reduced concentration of bifidobacteria and lactobacilli. The degree of manifestation of dysbiotic rearrangement of microflora largely depends on the initial state of the microenvironmental status.

N.N. Lizko’s discussed the type of stress-induced changes in his review [13]. Stressful situations establish the conditions for modification of the adhesive properties of bacteria and cell adhesiveness of a host. For example, the physical and chemical state of intestinal mucin may be disrupted by bile acids, proteolytic enzymes, and pH changes. An abrupt reduction in the mucosal component (mucin) and a decreased level of acidic mucopolysaccharides on the surface of the mucosal layer and mucosal lining cells are considered to be indicators of reaction to stress. Direct evidence support the existence of a certain predilection for changes in adhesion during stress-induced shifts in the digestive process. Notable changes in immune reactivity were observed in response to stress-induced activation of the hypothalamic-pituitary-adrenal axis. Reduced immune resistance can affect the topographic distribution of some microbial populations in the GIT. This may result in endogenous contamination and metabolic consequences of enhanced bacterial growth in the small intestine.

Thereby, disruption of the colonization resistance syndrome develops in almost all cases of humans living in artificially modified habitats. However, development of this syndrome depends on both specific factors, e.g. altered habitat factors (space radiation, micro-gravity, hypokinesia for space flights; a combination of an altered gaseous environment and changes in the pressure for hyperbaric conditions, etc), and non-specific factors, primarily stress-induced factors, and factors related to an enclosed space. They affect almost all colonization barriers.

**Fig. 1:**
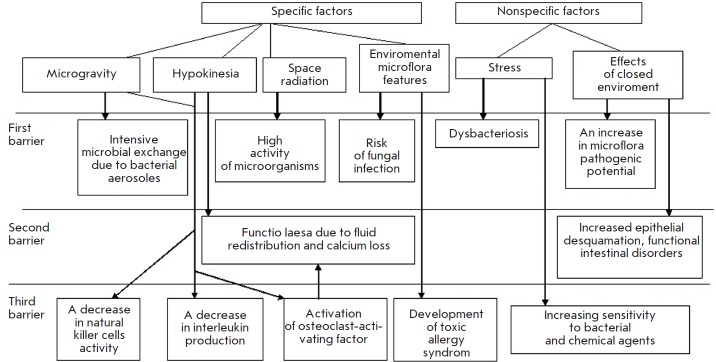
Scheme showing the development of the colonization resistance disruption syndrome in space flights

Thus, during a space flight (*Fig. 1*), a combination of specific (microgravity, hypokinesia, space radiation, colonization of environmental elements by bacterial– fungal associations) and non-specific factors (stress, closed environment factors) affect the conditions of all three colonization barriers. Intensive microbial exchange, exogenous contamination, stress-induced dysbacteriosis, and increased pathogenic potential in the human-microorganism system lead to a weakening of the first barrier formed by protective microflora. The second barrier (epithelial tissue and mucosa) also experiences a weakening of its protective properties due to several pathophysiological processes (redistribution of fluids, disruption of calcium homeostasis, increased desquamation of epithelium, and disruption of the physiological function of the intestine). The third barrier (factors of cell-mediated and humoral immunity) is also impaired, thus leading to changes in phagocytosis, serum bactericidal activity, reduced activity of killer cells and decreased production of interleukins, activation of the osteoclast-activating factor, and development of toxic and allergic conditions.

**Fig. 2:**
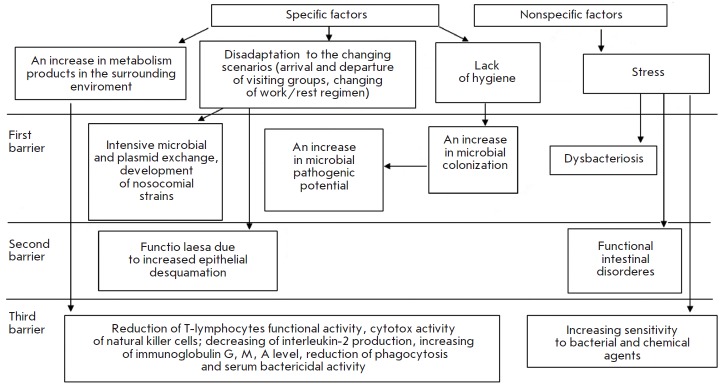
Scheme showing the development of the colonization resistance disruption syndrome in ground-based closedchamber studies

In inhabited ground-based closed-chambers (*Fig. 2*), specific environmental factors (elevated concentration of metabolic products, deadaptation, limits on hygienic procedures) and stress promote an increased intensity of both microbial and plasmid exchanges, which results in the spontaneous formation of nocosomial- like strains as early as during the first days of isolation, as well as an increased size of microbial foci, replacement of less virulent strains with more virulent strains of the same species, systemic dysbacteriosis, etc. The physiological status of epithelial tissue is also affected. The disrupted immunity manifests itself in a reduced functional activity of T lymphocytes and cytotoxic activity of natural killer T cells; a decreased production of interleukin 2; increased levels of immunoglobulins A, M, G; weakened phagocytosis and blood serum bactericidal activity; and increased sensitivity to bacterial and chemical agents.

**Fig. 3:**
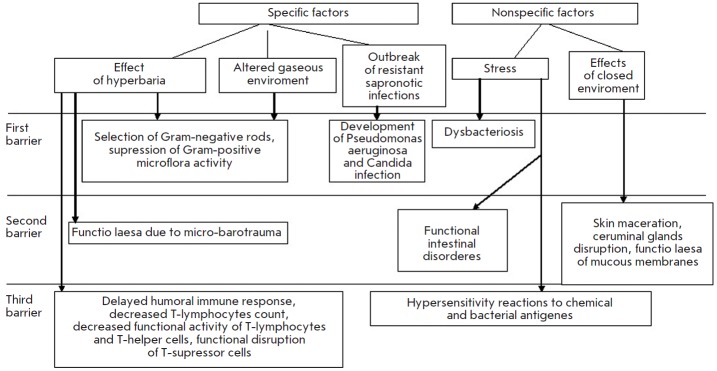
Scheme showing the development of the colonization resistance disruption syndrome under hyperbaric conditions

Disruption of the colonization resistance syndrome caused by a prolonged exposure to hyperbaric conditions is the most pronounced and most dangerous one (*Fig. 3*). Under such conditions, a combination of specific factors (a combination of the influences of a hyperbaric environment and altered gaseous environment) leads to pronounced activation of opportunistic microflora and suppression of the protective microflora. As a result, a manifestation of the disruption of the colonization resistance syndrome develops linearly with intensive colonization of the habitat with sapronose infectious agents, mainly *Pseudomonas aeruginosa*. The severity of the syndrome depends on the length of stay in hyperbaric conditions and the pressure. The barrier functions of epithelial tissue are additionally impaired by micro-barotraumas, maceration of epithelium, a decreased activity of ceruminal glands in external auditory canals, which become *locus minoris resistentiae *for infections. Infection caused by prolonged diving often manifests itself as external otitis, which results in premature decompression of the affected divers for medical reasons (*Table 1*) [7].

**Table 1:** Development of the disruption of colonization resistance under hyperbaric conditions, presented as a change in ratios between the protective and opportunistic microflora in epithelial tissue and the intestine

Stage	Day of study	Group of microorganisms, %		
		Protective Gram-positive	Opportunistic Gram-negative	Pseudomonas aeruginosa
Baseline	0	90.3	4.8	0
The isopression phase	1–3	67.3	11.5	9.6
	5-8	62.5	32.8	25.0
	11-13	39.6	31.0	20.6
Decompression	18-22	25.0	31.0	25.8
	24-28	38.7	40.3	29.0
Yield	30-34	68.5	68.5	22.8

## METHODS FOR PREVENTING THE DISRUPTION OF THE COLONIZATION RESISTANCE SYNDROME

Various commercial probiotic preparations based on collection strains are widely used to prevent and treat dysbacterioses of different origins. However, it is well known that probiotics introduced into the body may create an imbalance in the host’s autoflora as a result of antagonism between the indigenous and industrial strains [14]. Some researchers have shown that probiotics based on industrial strains fail due to biological incompatibility [15]. The number of reports on the adverse effects of probiotic therapy with preparations containing industrial strains is on the rise. Most papers report infectious complications associated with the use of commercial probiotics. Such adverse effects are likely to develop in weakened, elderly or immunecompromised patients. This fact should always be taken into account when using probiotic correction measures.

Detailed data on the complications associated with probiotic therapy of both bacterial and fungal etiology is presented in review [16] and partially summarized in *Table 2*.

**Table 2:** Cases of bacterial and fungal sepsis chronologically associated with the use of probiotics^*^

Form of sepsis	Patient’s age (years	Risk factors	Probiotic	Identification method	Reference
Hepatic abscess	74	Diabetes	LGG**	API 50 CH***, PFGE	[17]
Endocarditis	67	Mitral insufficiency, tooth extraction	*Lactobacillus rhamnosus,* 3 × 10^9^ CFU/day	API 50 CH, mass spectrometry	[18]
Bacteraemia	11 months	Prematurity, gastrostomy, short bowel syndrome, CVC, intravenous feeding, rotavirus-induced diarrhea	LGG, *1/4 capsule/day*	rRN A sequencing	[19]
Endocarditis	4 months	Cardiac surgery, antibiotics-associated diarrhea	LGG, *10^10^ CFU/day*	DNA dactylography	[20]
Bacteraemia	47	Not given	*Bacillus subtilis, 8 × 10^9^ spores/day*	Antibiotics sensitivity	[21]
Bacteraemia	73	Chronic lymphatic leukemia	B. subtilis, 8 × 10^9^ spores/day	16S rRN A sequencing	[22]
Fungaemia	3 months	CVC, diarrhea, intravenous feeding	*Saccharomyces boulardii,* 100 mg/day****	PFGE of mitochondrial DNA	[23]
Fungaemia	51	Chronic lymphatic leukemia	*S. boulardii,* 1 g/day	PFGE	[24]
Fungaemia	42	Kidney and pancreas transplantation, immunosupression, diarrhea associated with *Clostridium difficile*	*S. boulardii*, 1 g/day	PFGE	[25]

Notes. CVC– central venous catheter, rRNA – ribosome RNA, PFGE – pulsed field gel electrophoresis, LGG – Lactobacillus rhamnosus GG, CFU– colony-forming unit.

*Ref.[16]

**If the dosage is not listed in the table, the exact dosage was not provided in the original paper.

***A kit for identifying Lactobacillus spp., BioMerieux.

****250 mg of S. boulardii = 5.425 × 10^13^ living cells.

In addition to meeting the standards of genetic safety and resistance to antibacterial agents, the efficiency of a probiotic is also determined by its adhesive activity, lack of competitive relationships with the indigenous microflora, as well as by its antagonistic properties against opportunistic pathogens, which can be, to an extent, achieved through personalized selection of probiotic preparations [26]. The highest degree of preparation customization can evidently be reached by using autostrains of microorganisms selected from the microbiocenosis of the subject.

According to B.A. Shenderov, a fetus develops immunological tolerance to the normal microflora of the mother already during intrauterine growth [27]. According to the published data, the differences in the ability of industrial and autostrains to adhere to epithelium cells depend on the compatibility between a strain’s receptors and cell receptors [28-30]. Thus, it has been shown that vaginal lactobacilli adhere to cells of vaginal epithelium better than strains isolated from other sources [28, 29].

B.A. Berdichevsky studied the protective role of autobacteria under stress conditions [8]. Activation of the autochtonous microflora in response to surgical stress, which included translocation of autobacteria into extrainterstinal biotopes followed by elimination through the urinary tract, was demonstrated experimentally.

Some authors have proposed to use full human microbiocenosis as preparations [31-33]. To ensure infinite preservation of microflora it is recommended to store the biological material in cryobanks and subsequently use it to construct autoprobiotics and products of functional nutrition [31, 32]. The patent “Method for establishing a bank of autochtonous strains of microorganisms for the restoration of human intestine microbiocenosis” describes a method for producing a preparation of autolougous intestine microorganisms [33]. The method includes collecting stool samples from the same subject during clinically healthy periods, starting with days 7–15 after birth and subsequently at least once a year over his entire life. Autostrains of the normal intestine microflora are isolated from the samples and identified. The biomass of each bacterial species is grown on selective media up to at least 10^3^–10^9^ cells/ml. The resulting biomasses are joined and supplemented with a stabilizing solution. The mixture is divided into samples. Each sample is preserved during the entire lifetime of the subject with the biotiter being occasionally inspected. In infants less than 1 year old, the normal intestinal microflora predominantly consists of bifidobacteria* Bifidobacterium bifidum, B. brevis, B. infantis *and lactobacilli *Lactobacillus acidophilus, L. fermenti*. The normal intestinal microflora of individuals older than 1 year predominantly consists of bifidobacteria* B. longum, B. adolescentis*, lactobacilli *L. acidophilus, L. fermenti, L. plantarum*, strains of *Escherichia coli* and lactic streptococci *Streptococcus faccium, Str. faecalis, Str. avium, Str. salivarius *and *Str. bovis*. During the storage, equal amounts of autostrains samples isolated during different stages of a person’s life are further combined after biotiters are inspected. The introduction of a sample from the bank of autostrain microorganisms into the intestine makes it possible to simultaneously affect different components of the disrupted intestine biocenosis due to the use of the entire range of normal intestinal microflora [33].

The method for obtaining an autoprobiotic culture based on enterococci was described in patent “Method for obtaining autoprobiotic based on *Enterococcus faecium,* a representative of the indigenous microflora of a host intestine” [34]. The novelty of the proposed method for preparing lactic culture lies in the fact that the product is prepared from one’s own (autoprobiotic)* E. faecium *strain isolated from his gastrointestinal tract. This method proposed for the first time to use the polymerase chain reaction (PCR ) after the selection of colonies with a characteristic morphology and directly before preparing the product in order to determine the entoroccocus species and genetic determinants of pathogenicity. This approach guarantees absolute safety of the end product for the subject. The method is also characterized by fast production of a mature culture (4–6 days following the day the material is collected). Isolation of indigenous enteroccoci from feces to produce autoprobiotic lactic acid cultures requires implementation of the following algorithm: to plate bacteria from the individual’s feces on a selective medium, to identify the genus to which the strain belongs, to run PCR to determine the enteroccocus species and potential pathogenicity factors, to select clones that satisfy genetic safety and physiological functionality criteria, to store individual entercoccocus strains in a cryobank, and finally to produce the lactic culture [34].

In 2006, Van Likui comprehensively studied the treatment of bacterial vaginosis with autochtonous microorganisms [35]. The use of a commercial lactobacterin preparation in the form of a vaginal suppository improved the content of the vaginal microflora; however, it did not result in full recovery of lactic microflora due to the low survival rate of lactobaccilli from the intestine in the vagina. It was proposed to use autochtonous lactobacilli isolated from female patients’ vaginas to correct microbiocenosis impairment. Suppositories containing lactobacilli autostrains were topically applied after a course of antibacterial therapy, which ensured successful implantation of probiotics and significantly reduced the risk of recurrent bacterial vaginosis. A similar method was successfully employed to restore the disrupted microbiocenosis of vaginas in pregnant women after treating pyelonephritis with antibiotics [36].

It was demonstrated [37] that the commercial bacterial preparation Acylact is quickly eliminated from the vaginal environment. A 12-month-long randomized double-blind trial of commercial and autologous lactobacilli was carried out. A total of 165 women with bacterial vaginosis were involved in the study. One hundred and thirty-two patients underwent control examination: 70 patients from the group that received autochtonous lactobacilli (group I) and 62 patients from the group that received the Acylact preparation (group II). The therapeutic efficiency was measured using the criteria of clinical response, recovery of vaginal microbiocenosis and disappearance of the objective symptoms accompanying the disease. Prevention efficiency was measured by the number of relapses over the entire follow-up period. The dynamics of vaginal biocenosis recovery to its normal state was significantly different between the two treatment methods. When using autochtonous lactobacilli, biocenosis in most women (82.2%) recovered its normal state as early as after 3 months, while the maximum values (88.7%) were observed after 6 months; at the end of the study, the improvement was only 1.5%. When using Acylact preparations, the recovery to the normal state after 3 months was observed only in 61.7% of patients; after 6 months, in 71.4% of patients; and after 12 months, in 78% of patients. The rate and percentage of recovery in group II were significantly different from the values observed for group I (*p *≤ 0.05). The results of these studies show that lactobacilli exhibit genetic heterogeneity, which defines its specificity towards the host [37].

## CONCLUSIONS

The use of indigenous strains of protective microorganisms to correct dysbiosis has been studied by several authors [31-43]. The application of autostrains to prevent and treat dysbacteriosis in people holding extreme jobs (astronauts, pilots, divers, athletes, rescue workers) is particularly relevant. The lack of infectious complications, biological incompatibility and low acceptability make it possible to suggest that these preparations can be used to treat newborns, elderly people, and patients with a suppressed immune system. The concept of creating cryobanks of human microbiocenosis and autoprobiotics can be considered at the moment as a separate branch of personalized medicine. A customized approach to drug selection allows one to improve the efficiency of both prevention and treatment, as well as to reduce the risk of adverse drug reactions in each patient.
